# Assessment of Hammocks (Petenes) Resilience to Sea Level Rise Due to Climate Change in Mexico

**DOI:** 10.1371/journal.pone.0162637

**Published:** 2016-09-09

**Authors:** Mariana C. Hernández-Montilla, Miguel Angel Martínez-Morales, Gregorio Posada Vanegas, Bernardus H. J. de Jong

**Affiliations:** 1 Departamento de Conservación de la Biodiversidad, El Colegio de la Frontera Sur, Campeche, Campeche, México; 2 Instituto de Ecología, Pesquerías y Oceanografía del Golfo de México, Universidad Autónoma de Campeche, Campeche, Campeche, México; 3 Departamento de Ciencias de la Sustentabilidad, El Colegio de la Frontera Sur, Campeche, Campeche, México; University of Vigo, SPAIN

## Abstract

There is a pressing need to assess resilience of coastal ecosystems against sea level rise. To develop appropriate response strategies against future climate disturbances, it is important to estimate the magnitude of disturbances that these ecosystems can absorb and to better understand their underlying processes. Hammocks (petenes) coastal ecosystems are highly vulnerable to sea level rise linked to climate change; their vulnerability is mainly due to its close relation with the sea through underground drainage in predominantly karstic soils. Hammocks are biologically important because of their high diversity and restricted distribution. This study proposes a strategy to assess resilience of this coastal ecosystem when high-precision data are scarce. Approaches and methods used to derive ecological resilience maps of hammocks are described and assessed. Resilience models were built by incorporating and weighting appropriate indicators of persistence to assess hammocks resilience against flooding due to climate change at “Los Petenes Biosphere Reserve”, in the Yucatán Peninsula, Mexico. According to the analysis, 25% of the study area is highly resilient (hot spots), whereas 51% has low resilience (cold spots). The most significant hot spot clusters of resilience were located in areas distant to the coastal zone, with indirect tidal influence, and consisted mostly of hammocks surrounded by basin mangrove and floodplain forest. This study revealed that multi-criteria analysis and the use of GIS for qualitative, semi-quantitative and statistical spatial analyses constitute a powerful tool to develop ecological resilience maps of coastal ecosystems that are highly vulnerable to sea level rise, even when high-precision data are not available. This method can be applied in other sites to help develop resilience analyses and decision-making processes for management and conservation of coastal areas worldwide.

## Introduction

There is evidence that climate change, in particular sea level rise (SLR), will have a significant impact on coastal ecosystems, specifically those that are already threatened by other anthropogenic disturbances, and Mexican coastal ecosystems are not an exception [1]. Nevertheless, their assessment of resilience to future climatic changes is often hampered by the lack of appropriate data and information [2]. This hinders the implementation of effective conservation strategies in these vulnerable environments.

**Fig 1:**
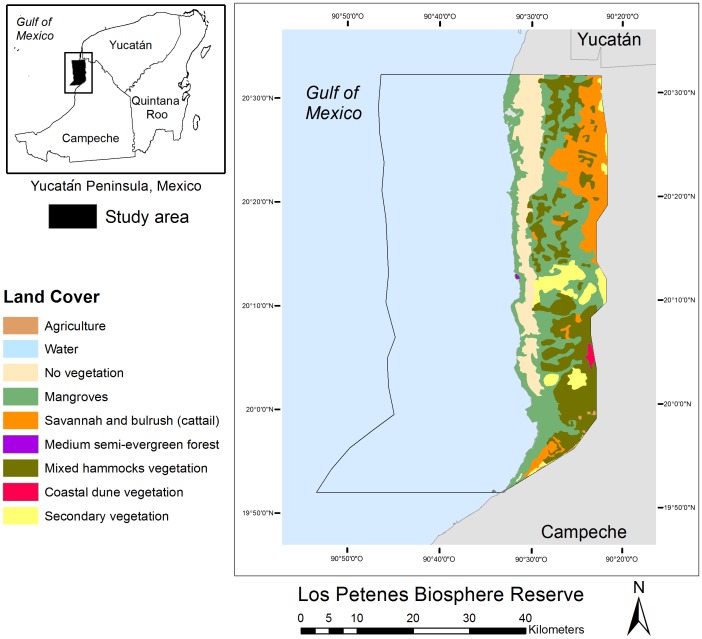
Location of Los Petenes Biosphere Reserve in the Yucatán Peninsula, Mexico.

According to the Fifth National Communication of Mexico to the UN Framework Convention on Climate Change of 2012, the sea surface temperature in the Caribbean, the Gulf of Mexico, and the Mexican Pacific may rise between 1 and 2°C by 2020; and in general, the climate of Mexico will be between 2 and 4°C warmer around 2050 [2]. This warming of sea water could trigger a SLR between 20 and 165 cm, as well as changes in rainfall, storm, and hurricane patterns, which would bring about the flooding of many cities and coastal areas in the country [3]. Under these scenarios, it is likely that coastal systems and low-lying areas will increasingly experience adverse impacts such as temporal and permanent submergence, flooding, and erosion [4,5]. Hammocks (petenes) are one of the most vulnerable coastal ecosystems [6,7]. This unique ecosystem is a highly diverse species assemblage linked to spring water holes; therefore, one of its main threats is the intrusion of saltwater into the freshwater aquifer due to SLR [8]. Despite its ecological importance and vulnerability, there are very few studies related to its resilience to disturbances. Hammocks are restricted to the Yucatán Peninsula in Mexico, the Everglades in Florida, and the Ciénaga de Zapata in Cuba [6,9,10]. In Mexico, hammocks are distributed in the north-western coast of the Yucatán Peninsula. In 1999, this region was decreed a natural protected area (NPA), “Los Petenes Biosphere Reserve” (LPBR). LPBR is a long, narrow coastal strip covering an area of 2,829 km^2^ in two zones: a land area of 1,009 km^2^ and a marine portion of 1,819 km^2^ (Fig 1).

Even though ecosystems and species can be protected from human disturbances in NPAs, they will not be exempt from natural disturbances due to climate change [11]. The Convention on Biological Diversity (CBD) encourages the development of tools and methods to aid countries to evaluate climate impacts on NPA systems and increase their resilience, by focusing on the mitigation and adaptation of the most vulnerable ecosystems [12]. Thus, resilience has become a key issue to assess the persistence of natural systems. This fact has allowed the generation of a growing number of policies aimed at buffering ecological systems from future large-scale disturbances [13–15].

Four decades ago, Holling introduced the term resilience in the ecological literature to explain the non-linear dynamics observed in disturbed ecosystems, by focusing on the persistence of populations or communities at the ecosystem level [16]. He defined ecological resilience as the amount of disturbance that an ecosystem can withstand without changing self-organized processes and structures [17]. Since then, multiple meanings of resilience have appeared in the literature, giving the concept a rich history, sometimes with a considerable stretch from its original meaning [18,19]. Some authors recognize two distinct and measurable components of response to disturbance: ‘Resistance’ as the ability to persist during the disturbance, and ‘recovery’ as the capacity to ‘bounce back’ following alleviation of the disturbance [15,20,21]. Both terms are merged within the concept of ‘ecological resilience’ [15,22]. Recognizing the dynamics of ecosystems, current resilience theories envision ecosystems as constantly changing. As a result, resilience is often evaluated in terms of the amount of change a given system can undergo and still remain within a set of natural or desirable states [23]. In this study, the concept of ecological resilience is defined as the magnitude of disturbance that hammocks can absorb before changes of states or tolerances occur in response to perturbations [24,25]. To make this concept practical and measurable, it needs to be applied to empirical cases, for which the dynamics of the ecosystem needs to be understood [26,27].

In this study, we use the vulnerability framework applied in the Risk Hazard (RH) model [23] to understand the impact of a particular hazard event, such as SLR, and the dose-response of the entity exposed (hammocks). The RH model was developed to make vulnerability analysis consistent with the concerns of sustainability and global environmental change science [23,28]. Quantitative applications of this model applied in environmental and climate impact assessments emphasize the importance of including exposure and sensitivity to perturbations and stressors in the analysis [23]. This study uses persistence of hammocks in response to SLR to assess the ecological resilience of the system [29], that in turn is derived from the core idea that multiple stability domains and equilibria may exist within an ecological system [30]. Persistence represents a fundamental property of the stability concept that corresponds to whole ecosystems; therefore, it is a holistic and qualitative concept of resilience [30]. As such, persistence of coastal ecosystems is determined by the ability of hammocks to cope totally or partially with SLR by growing vertically, migrating inland or expanding laterally [31].

In this study, a matrix is developed to assess hammocks resilience, based on measures and metrics of hammocks exposure and sensitivity to SLR due to climate change at different spatiotemporal scales. Exposure, sensitivity, and persistence are used to build a model that operationalize the resilience analysis, using a modified vulnerability framework of the RH model [23]. In the model, a resilient ecosystem has a low exposure and a low sensitivity to stressors, and thus experiences a high persistence. Exposure to stressors is therefore proportional to sensitivity and both are the inverse of persistence. This study uses an indicator-based approach to evaluate coastal vulnerability and to characterize aspects or the state of coastal ecosystems, such as drivers of change, pressure on the ecosystem, human impacts on the vegetation, exposure, sensitivity, and risk of flooding due to SLR [32]. The method is applied to a specific dynamic ecosystem, but can be readily adapted to other circumstances in data-poor scenarios.

## Methods

All field permits issued for conducting this research were obtained through the Direction of the LPBR of the Mexican Commission for Natural Protected Areas (CONANP), the state authority responsible for this NPA. Fieldwork did not involve collecting or damaging endangered or protected species.

### Resilience conceptual framework

A resilience conceptual framework was developed combining literature review, attendance at meetings of practitioners in the field of vulnerability, adaptation, resilience, and natural hazards, and also discussions with key individuals. Within the context of this framework, a set of indicators was developed to assess hammocks persistence to SLR, and thus, the resilience of this ecosystem.

**Fig 2:**
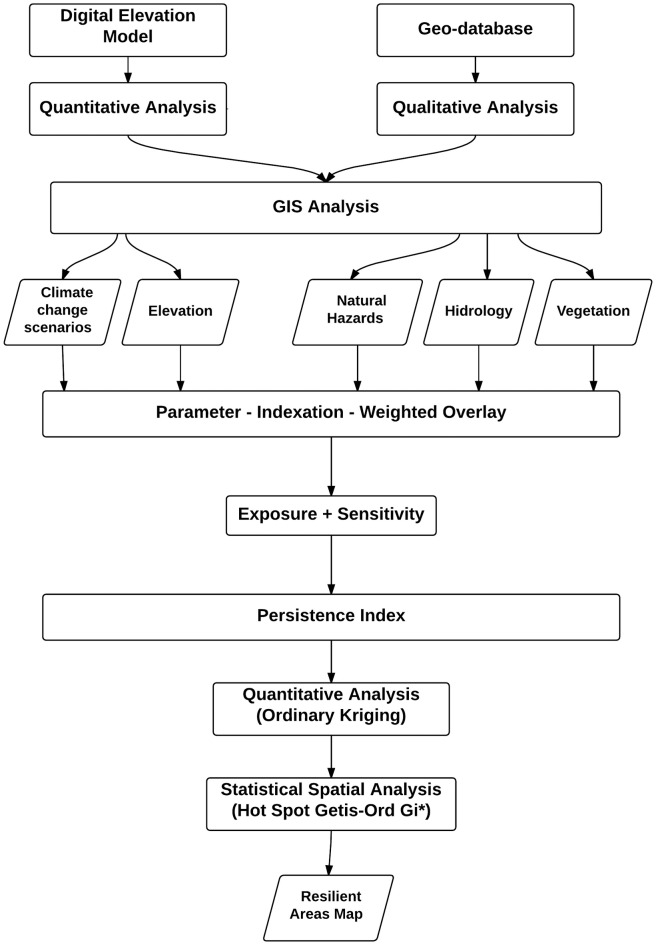
Flow chart of the methodology used to assess hammocks resilience to sea level rise due to climate change and to predict the most resilient areas at Los Petenes Biosphere Reserve.

The vulnerability analysis framework of the RH model [23] was adapted to our study, specifically to emphasize exposure and sensitivity to perturbations and stressors, considering persistence as part of the concept of ecological resilience. A hierarchical model was developed to measure, analyse, and weight the main ecological indicators to assess exposure and sensitivity criteria. The interaction of these criteria generated an index of persistence that was used to determine the most resilient areas of the LPBR (Fig 2).

### Data acquisition

Different digital data sources were assembled as GIS layers (Table 1). The GIS analyses were performed using ArcMap 10.0 (ESRI, Redlands) and IDRISI Selva (Clark Labs, Worcester). All data were georeferenced to a Universal Transverse Mercator (UTM) projection with the origin in zone 15N and the projected coordinate system WGS84 for the horizontal and vertical datum.

**Table 1:** Characteristics of the spatial data used.

Data	Scale	Date	Source
Aerial photographs	1:75,000 23 x 23 cm	Feb/1998	INEGI database <www.inegi.org.mx>
Landsat images (Landsat 2, 4, 7, 8)	Multiple scales Resolution: 30 m Path/Row: 21/46	Feb/1979, Apr/1990, Mar/ 2003, and Dec/2014	El Colegio de la Frontera Sur
SPOT 5 images (multispectral and panchromatic)	From 1:100,000 to 1:15,000 2.5 to 5 m in panchromatic mode 10 m in multispectral mode	Dec/2012	El Colegio de la Frontera Sur
Hydrological data and maps “HYDROSHED”	Multiple scales Resolution: 90 m	Feb/2000	Conservation Science Program of the World Wildlife Fund <www.worldwildlife.org/hydrosheds> or <hydrosheds.cr.usgs.gov/index.php> [33]
Digital Elevation/Surface Model (DSM)	• Multiple scales	2001 to present	NEXTMap World 30 m Digital Elevation/Surface Model data from Intermap Technologies [34,35]
Shapefiles of rivers and water bodies	• Multiple scales		INEGI database [36]
Mexican National Cartography on Land Use and Vegetation (Series V)	1:250,000 27.5 m per pixel data from: Landsat TM 30 m	2011	INEGI database [36]
Hurricane historical data V 4.0	• Multiple scales	1842–2013	IBTrACS (maps of storm tracks) International Best Track Archive for Climate Stewardship. NOAA National Climatic Data Center <www.ncdc.noaa.gov/ibtracs/index.php?name=browse> [37]

### Criteria assessment

Exposure and sensitivity to perturbations and stressors were defined as criteria to assess hammocks resilience and a set of indicators was identified to assess each criterion. A score from 1 to 3 was used to assess each indicator, where lower values represented less exposure or sensitivity. A total of 425 hammock polygons were delimited in the LPBR by analysing SPOT multispectral and panchromatic images, aerial photographs, and land use and vegetation maps. The indicators of both criteria were assessed for each of these polygons.

#### Exposure

Five indicators were used to assess exposure: Sea flooding due to SLR scenarios, coastal flooding, proximity to streams, hurricanes impact, and land cover change. The main underlying force in the first four indicators is the intrusion of saltwater due to SLR. Although tidal flooding could also be a stressor in the persistence of hammocks, it operates at fine temporal scales; in studies encompassing broader temporal scales, such as this, it is difficult to assess its effects. However, tidal flooding is an intrinsic risk factor in some of the indicators we defined to assess the exposure (e.g., hurricanes impact) and sensitivity (e.g., type of hammock) criteria.

**Sea flooding due to SLR scenarios**: Given the uncertainties on SLR projections due to climate change and based on the precautionary principle, this indicator was assessed using three SLR scenarios for the next 100 years. These scenarios were based on the estimations given in the Fourth and Fifth Assessment Report (AR4, AR5) of the Intergovernmental Panel on Climate Change (IPCC) as well as other regional projections [6, 38–40]. The mild scenario assumed an increase from 0 to 1 m above current sea level (ACSL), a medium scenario assumed an increase from >1 to 2 m ACSL, and the harsh scenario assumed an increase from >2 to 3 m ACSL.

A Digital Surface Model (DSM) was used to perform a series of GIS analyses to assess this indicator. The DSM was reclassified adjusting the outliers; then, elevation scores were assigned from 1 to 3, where 1 represented the values from >0 to 1 m above sea level (ASL), 2 from >1 to 2 m ASL, and 3 from >2 to 3 m ASL. Elevation values <0 m and >3 m were masked. The 425 hammock polygons were overlaid on the reclassified DSM to estimate the proportion of flooded area of each polygon in each of the three SLR scenarios. The categories of flooding due to SLR were assigned to the polygons according to their proportion of flooded area: (1) from 0 to 30%, (2) from >30 to 60%, and (3) >60%.

**Coastal flooding**: This indicator was considered because of the intrinsic vulnerability to sea flooding of areas with sinks and next to the coast. Three risk categories were defined for this indicator based on the hydrologic conditioning, the sink identification layer from HydroSHEDS, and the DSM. With this data, the low-lying areas close to the coastline and the natural sinks or depressions were delimited obtaining three zones that represented different exposure scores to sea flooding due to SLR scenarios. The most exposed zone (score 3) was the lowest-lying area (-5 to 3 m ASL) characterized by jungle hammocks, located in the coastline (<4 km from the coastline) and coastal lagoons [41]. Conversely, the less exposed zone (score 1) corresponded to the highest zones (>8 m ASL) and the areas farthest from the coastline (>6 km from the coastline). A score of 2 was assigned to the remaining areas.

These three risk zones were intersected with the 425 hammock polygons layer to assign a risk value to each polygon. Since some of the hammock polygons were intersected by two risk zones, the risk category assigned was based on the risk zone with the largest proportion of area and the average value of the neighbouring polygons.

**Proximity to streams**: Sea flooding due to SLR may be facilitated with the presence and proximity to streams. Modelling the hydrodynamic flow of watercourses was difficult because of the lack of high precision data and logistical constrains to make accurate measurements at the LPBR. Therefore, based on discussions with specialists, three distance buffers from streams (0–100, >100–500, >500 m) were created. These buffers were intersected with the 425 hammock polygons layer in order to assign an unsupervised risk category to each polygon (from 0 to 3). Zero represented no intersection, (1) >500 m from streams, (2) from >100 to 500 m, and (3) within 100 m from streams. Again, when a polygon was intersected by more than one risk category, it obtained the category with the largest proportion of area.

**Hurricanes impact**: Hurricanes are a disturbance factor that may potentially affect hammocks persistence due to storm surges and vegetation damage. Hurricanes in the Atlantic coast can cause storm surges of 5 m or more [42]. The intensity and frequency of Atlantic hurricanes have increased substantially in recent decades. Based on numerical models [38], these phenomena are likely to become more intense as surface temperature of tropical sea increases [42]. Assessing this indicator relied on previous information of hurricane occurrence in the region through the analysis of hurricane frequency and intensity, as well as its relationship with terrain. First, historical data of hurricane occurrence within a buffer of 200 km from the LPBR were downloaded from the NOAA website [37] in GIS format. A total of 990 hurricane tracks were retrieved over a period of 171 years (1842–2013). A buffer for each hurricane track was generated according to its category (Saffir-Simpson scale) and inland effect [43,44]. Based on its intensity (tropical depression [TD], tropical storm [TS], and hurricane [H] from 1 to 4), the width of the buffer was defined as: TD = 1.6 km, TS = 3.2 km, H1 = 6.4 km, H2 = 9.7 km, H3 = 12.9 km, and H4 = 16.1 km [44].

The buffers and the hammock polygons layers were intersected, obtaining a total of 485 tracks that directly hit the LPBR. The number of times that each hammock polygon was intersected by a hurricane and its type was estimated. Each hammock polygon was assigned a value by adding the products of the number of hurricanes that hit it by their intensity. These values were then normalized from 1 to 3 in order to assign a hurricane risk category to each hammock polygon.

**Land cover change**: Land cover change (LCC) due to human activities affects the persistence of native land cover. To assess this indicator, the differences in reflectance between forested and non-forested areas were discerned using four georeferenced and orthorectified cloud-free Landsat scenes at 30 m resolution (Table 1). A combination of unsupervised classification methods was used to digitally classify the pixels in all four Landsat images with the Iterative Self-Organizing Data Analysis Technique (ISODATA) in Idrisi Selva 17.0 (Clark Labs, Worcester). Six thematic classes were distinguished, which represented the land cover types in the study area, excluding shadow and cloud cover. The land cover types were: (1) high and dense vegetation mainly of hammocks and mangroves, a class whose pixels were very green with the highest Normalized Difference Vegetation Index (NDVI) values characterized by the homogeneity of forest cover; (2) discontinuous secondary forest, a forested class with low-density vegetation whose mixed pixels are characterized by a variety of land cover types including hammocks, intervened vegetation, low mangroves, and secondary mixed forest with a relatively high NDVI value; (3) bushes and shrubs whose pixels have a low to medium NDVI values; (4) coastal dune scrub communities, cattail, grass, and brush whose pixels have a low NDVI; (5) bare soil or sparse vegetation and dry grass, and (6) water (Table 2). Since images from different sensors and dates were used in the analysis, the NDVI values varied. For example; in the 1979 image, NDVI class 1 values ranged from 0.74 to 0.97, and in the 1990 image from 0.41 to 0.95; thus, NDVI class 1 had different values depending on the image date ranging from 0.32 (in 2014) to 0.97 (in 1979; Table 2).

**Table 2:** NDVI minimum and maximum values per class computed by authors based on Landsat imagery (1979, 1990, 2001, and 2014).

NDVI class	Land cover type	1979		1990		2001		2014	
		Min	Max	Min	Max	Min	Max	Min	Max
1	Hammocks Mangroves	0.74	0.97	0.41	0.95	0.47	0.69	0.32	0.51
2	• Secondary forest	0.31	0.74	0.15	0.41	0.31	0.47	0.21	0.32
3	Bushes Shrubs	-0.14	0.31	0.19	0.15	0.03	0.31	0.04	0.21
4	Coastal dune scrub Cattail Grass	-0.36	-0.14	-0.43	0.19	-0.19	0.03	-0.11	0.04
5	Bare soil Sparse vegetation	-0.63	-0.36	-0.99	-0.43	-0.53	-0.19	-0.35	-0.11

Pixels that could be clearly identified as belonging to one of the six classes were extracted from the image to reduce spectral variability across the remaining pixels. Reflectance values corresponding to dense vegetation and exposed areas such as salt flats and crops were used to create spectral signatures for classes. The remaining pixels were grouped into the mixed class containing mangroves, secondary vegetation, and small-scale subsistence agriculture. This process was applied to each Landsat scene. A GIS-based spatial tool was used to assign pixels to the most likely thematic class based on their location to discriminate among similar classes and to avoid classification errors in established classes. No field data were available, so secondary reference data were used to validate map accuracy; for example, the national vegetation maps were used to discriminate among different types of forest and the orthophotos helped in identifying the agricultural class.

Land conversion analyses were performed with the module Land Change Modeler available in Idrisi Selva [45]. The software provides a rapid assessment of quantitative change by graphing gains and losses per category or it examines the contribution to changes experienced by a single land cover [45]. In these analyses a number of predictors were considered including slope, distance to settlements, distance to roads, and distance to agriculture to calibrate the model. Transitions smaller than 1 km^2^ were ignored and a cross classification was used to compare the transition between the four classified images. The proportion of LCC in each hammock polygon was assessed.

Scores were assigned to each polygon according to the transition categories from 1 to 3, where 1 represented recovery (an increase in the NDVI value), 2 perturbation (a decrease in the NDVI value of one or two class levels), and 3 the loss of native vegetation (a decrease in the NDVI value of more than two class levels; Table 3). The polygons that showed no transition were also assigned a score of 1. The overall transition score per polygon was calculated by averaging the transition scores of its pixels.

**Table 3:** Transition scenarios of land cover type categories based on NDVI classes using Land Change Modeler.

Transition	Land cover change score	Land cover change scenario (NDVI class)
Recovery	1	5, 4, 3, 2 to 1 / 4 to 3, 2 / 3 to 2
Perturbation	2	4 to 5 / 3 to 4, 5 / 2 to 3, 4 / 1 to 2, 3
Loss	3	1, 2 to 5 / 1 to 4

#### Sensitivity

Sensitivity was defined as the intrinsic potential of hammocks to be affected by SLR given their features. To assess this criterion, satellite imagery was used to estimate hammocks structural properties. For this criterion three indicators were defined: Hammock canopy height, type of hammock, and patch size.

**Hammock canopy height**: This indicator assumed that canopy height is a proxy of habitat quality and that habitat quality influences the vulnerability of hammocks to SLR. A calibrated high-resolution (30 m) DSM was used to measure forest canopy height [46]. The DSM and the hammock polygons layer were overlaid to assign a score to polygons based on their canopy height. Mangrove hammock forest scored 1. This type of forest has an average height of 15 m or more due to the high concentrations of nutrients, low salinity, and soil with a thick layer of organic matter. They are associated to spring waterholes. *Rizophora mangle* and *Laguncularia racemosa* are the dominant species [41]. Medium height mixed forest scored 2. Canopy height in this forest is between 3 and <15 m. This category includes deciduous forest, floodplain forest, and lowland deciduous forest among others [41]. Finally, the dwarf mangrove forest scored 3, where canopy height is between 1.5 and <3 m. This forest develops in nutrient limited sediments and high salinity. It is dominated by *Rizophora mangle* [41]. Some hammock polygons were intersected by two canopy height categories, therefore the final score was based on the category which covered the largest proportion of the polygon.

**Type of hammock**: Each type of hammock has a specific vulnerability to SLR. Three types of hammocks were identified and delimited using the orthophotos and the hammock polygons layer: basin hammocks, mixed hammocks, and fringe hammocks. Then the type of hammock was rated based on their intrinsic sensitivity to SLR, basin hammocks scored 1, mixed hammocks scored 2, and fringe hammocks scored 3.

Basin hammocks are located behind the coastline, the tidal influence is indirect and lower compared to the fringe hammocks. This community can be monospecific or a mixed species forest of *Avicennia germinans*, if salinity is high, and *Laguncularia racemosa*, if salinity is relatively low [41]. On the other hand, fringe hammocks are mainly located on the coastline and in coastal lagoons. This type of hammock is influenced by astronomical tides with semidiurnal trends with a maximum value of +0.49 m and a minimum of -0.72 m a.s.l. [47], so they are flooded and dried practically every day. In addition, they are exposed to winds and waves of up to 0.5 m, which can increase to more than 2.0 m during storms [47,48]. The dominant species is *Rizophora mangle* [41]. Some of the hammocks (mixed hammocks) could not be distinguished from basin and fringe hammocks; in this case, hammocks could be influenced both by winds and tides, but their impact is lower than in the fringe hammocks.

**Patch size**: Patch size is one of the most used metrics in landscape analyses [49] and size is known to be correlated with some measure of persistence or stability. The hammock polygons layer was used to classify hammocks by their size. Hammock patches ranged from 0.15 to 89.6 km^2^. It was assumed that larger patches were less sensitive to SLR; in contrast, smaller patches were relatively less likely to rebound from disturbances because they were more likely to be fragmented or destroyed after changes in hydrology, destruction of vegetation, or the establishment of opportunistic flora and fauna [50]. Three size categories were defined: The largest patches (>5 to 89.6 km^2^) got the lowest value of sensitivity (1), the smallest patches (>0.15 to 1.5 km^2^) got the highest value of sensitivity (3), and the remaining patches (>1.5 to 5 km^2^) got a medium value of sensitivity (2).

### Persistence

The interaction of climatic and anthropogenic factors influence the persistence of coastal ecosystems [51], together with their intrinsic vulnerability. In this study, hammocks persistence was defined by the interaction between exposure and sensitivity criteria. To assess hammocks persistence, each indicator of both criteria was weighted to express its importance relative to the other indicators, based on the pairwise comparison method [52]. A priority vector (weights) was estimated from the pairwise comparison matrix. The relative importance was based on the influence of each indicator in the flooding of hammocks, derived from the opinion of experts and published research. The relative importance ranges from 1 (equally important) to 9 (extremely more important). In the pairwise comparison matrix, relative importance values are allocated for the more important indicators against less important indicators, while the reciprocal values are allocated for the less important indicators against more important indicators (Tables 4 and 5). Since this study assumes that it is likely that SLR due to climate change will directly contribute to the increase in hammocks’ exposure, the assessment of the “sea flooding due to SLR scenarios” exposure indicator was assigned the highest value of relative importance, compared to the other exposure indicators. Conversely, the “hurricanes impact” exposure indicator was assigned the lowest value, based on the fact that even when a hurricane could affect both terrestrial and coastal margins, particularly increasing the mortality of trees with small diameters (3.3–10 cm) [53], a recent study states that other factors such as anthropic disturbances may have a larger and more permanent impact on hammocks in this area [54]. “Coastal flooding”, “proximity to streams”, and “land cover change” indicators were assigned the same relative importance because although LCC may have more permanent adverse effects on the persistence of hammocks, the other two indicators have a more extensive effect. In the case of sensitivity, the “type of hammock” indicator was rated with the highest value of relative importance, compared to the other two sensitivity indicators, since trees in the coastline are most sensitive to processes such as submergence, coastal flooding, and coastal erosion [4,5].

**Table 4:** Pairwise comparison matrix for exposure indicators.

**Indicator**	Sea flooding due to SLR scenarios	Coastal flooding	Proximity to streams	Land cover change	Hurricanes impact
Sea flooding due to SLR scenarios	1	2	2	2	3
Coastal flooding	1/2	1	1	1	2
Proximity to streams	1/2	1	1	1	2
Land cover change	1/2	1	1	1	2
Hurricanes impact	1/3	1/2	1/2	1/2	1

**Table 5:** Pairwise comparison matrix for sensitivity indicators.

**Indicator**	Type of hammock	Hammock canopy height	Patch size
Type of hammock	1	2	2
Hammock canopy height	1/2	1	1
Patch size	1/2	1	1

From the pairwise comparison matrices of both criteria, weights were calculated, as shown in Table 6.

**Table 6:** Weighting values applied to the scores of all the indicators of each criterion to assess hammocks persistence.

Criteria	Indicator	Weight
Exposure	Sea flooding due to SLR scenarios	0.35
Exposure	Coastal flooding	0.18
Exposure	Proximity to streams	0.18
Exposure	Hurricanes impact	0.12
Exposure	Land cover change	0.18
Sensitivity	Hammock canopy height	0.25
Sensitivity	Type of hammock	0.50
Sensitivity	Patch size	0.25

Persistence of each hammock polygon was estimated, based on the following expression:

$$P=\frac{1}{\left(E+S\right)}*100$$

where,

*P* = persistence index

*E* = the added weighted scores of all exposure indicators

*S* = the added weighted scores of all sensitivity indicators

A sensitivity analysis was performed to assess the sensitivity of the estimated hammocks resilience to the choice of alternative weighting schemes of indicators. Three weighting schemes were assessed, besides the baseline weighting scheme used (Table 7).

**Table 7:** Weighting schemes used to assess their effect on the estimated resilience of hammocks in the LPBR.

Criteria	Indicator	Baseline weighting	Scheme 1	Scheme 2	Scheme 3
Exposure	Sea flooding due to SLR scenarios	0.35	0.2	0.2	0.2
Exposure	Coastal flooding	0.18	0.2	0.2	0.3
Exposure	Proximity to streams	0.18	0.2	0.2	0.1
Exposure	Hurricanes impact	0.12	0.2	0.1	0.2
Exposure	Land cover change	0.18	0.2	0.3	0.2
Sensitivity	Hammock canopy height	0.25	0.3	0.4	0.3
Sensitivity	Type of hammock	0.50	0.3	0.3	0.3
Sensitivity	Patch size	0.25	0.3	0.3	0.4

### Data interpolation

Exposure, sensitivity, and persistence values calculated for each hammock polygon were interpolated by Ordinary Kriging to generate maps with five symmetric-range categories for each criterion (very high, high, moderate, low and very low). This technique is one of the most frequently used geo-statistical methods, and is quite efficient and accurate for spatial prediction and interpolation; it assumes that there is no constant mean for the data and area mean (i.e., no trend) [55].

### Delimitation of resilient areas

A spatial pattern detection technique was used to analyse clusters of high and low values of persistence. This analysis allowed to identify areas that experience a significantly higher level of persistence to coastal flooding due to SLR in the LPBR, and to assess that these values were not random events. The Getis–Ord G_i_* statistic [56] was used to assess such hot spots of resilience at the LPBR.

$$G_{i}^{*}=\frac{{\sum^{\text{​}}}_{j=1}^{n}w_{ij}x_{j}-\bar{X}{\sum^{\text{​}}}_{j=1}^{n}w_{ij}}{S\sqrt{\frac{\left[n{\sum^{\text{​}}}_{j=1}^{n}\;w_{ij}^{2}-{\left({\sum^{\text{​}}}_{j=1}^{n}w_{ij}\right)}^{2}\right]}{n-1}}}$$

where,

*x*_*j*_ = estimated persistence index value of polygon *j*

*w*_*ij*_ = spatial weight between polygon *i* and *j*. The conceptualization of the spatial relationships among polygons (spatial weight) was based on the fixed distance band method assuming Euclidian distances [55].

*n* = total number of hammock polygons

$$\bar{X}=\frac{{\sum^{\text{​}}}_{j=1}^{n}x_{j}}{n}$$

$$S=\sqrt{\frac{{\sum^{\text{​}}}_{j=1}^{n}x_{j}^{2}}{n}-{\left(\bar{X}\right)}^{2}}$$

This statistic generated a continuous variable of standardized intervals measuring the cold spots or very low resilient areas (-6.32 to -1.79 G_i_* score), low resilient areas (>-1.79 to 0.33), moderate resilient areas (>0.33 to 2.11), and high (>2.11 to 6.19) and very high resilient areas (>6.19 to 9.98), the hot spots of resilience. In this sense, G_i_* score close to zero shows no pattern (a random distribution), whereas a high G_i_* score shows a clustered structure and represents highly resilient areas.

## Results

**Fig 3:**
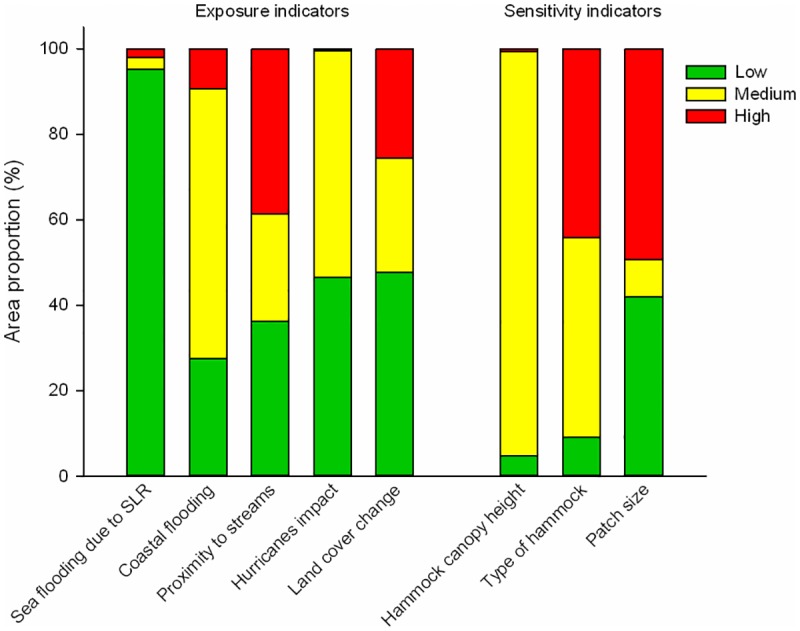
Area proportion in Los Petenes Biosphere Reserve of the risk level of each indicator of exposure and sensitivity criteria.

**Fig 4:**
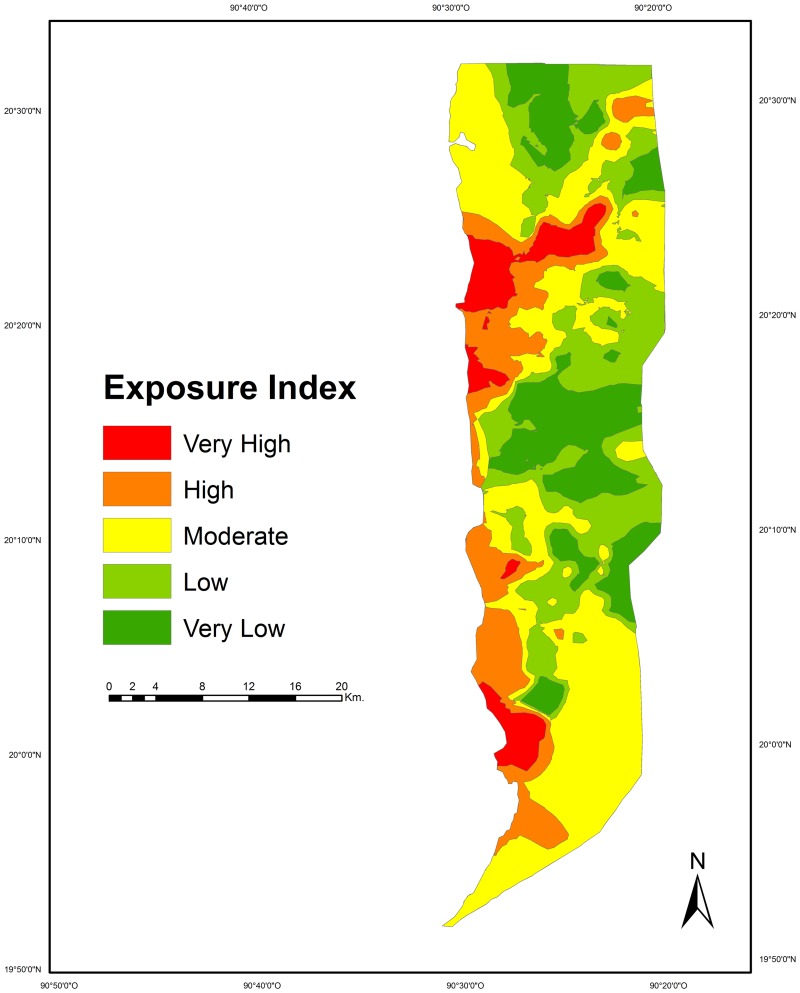
Map of exposure to sea level rise due to climate change at Los Petenes Biosphere Reserve.

The assessment of the exposure criterion showed that most of the LPBR has a low risk for the indicators “sea flooding due to SLR scenarios” (95.1% scored 1, 2.8% scored 2, 2.1% scored 3) and “LCC” (47.6% scored 1, 26.8% scored 2, 25.6% scored 3). On the other hand, a large proportion of the LPBR corresponded to the moderate risk category for the indicators “coastal flooding” (63.1% scored 2, 27.5% scored 1, 9.4% scored 3) and “hurricanes impact” (53.0% scored 2, 46.5% scored 1, 0.4% scored 3). Finally, for the indicator “proximity to streams” 38.5% of the LPBR corresponded to the high risk category (score 3), while 25.1% scored 2, and 36.3% scored 1. Based on the interpolation of the exposure indicators, 17.3% of the study area presented very low risk, 24.2% low, 37.5% moderate, 14.3% high and 6.7% very high (Figs 3 and 4).

**Fig 5:**
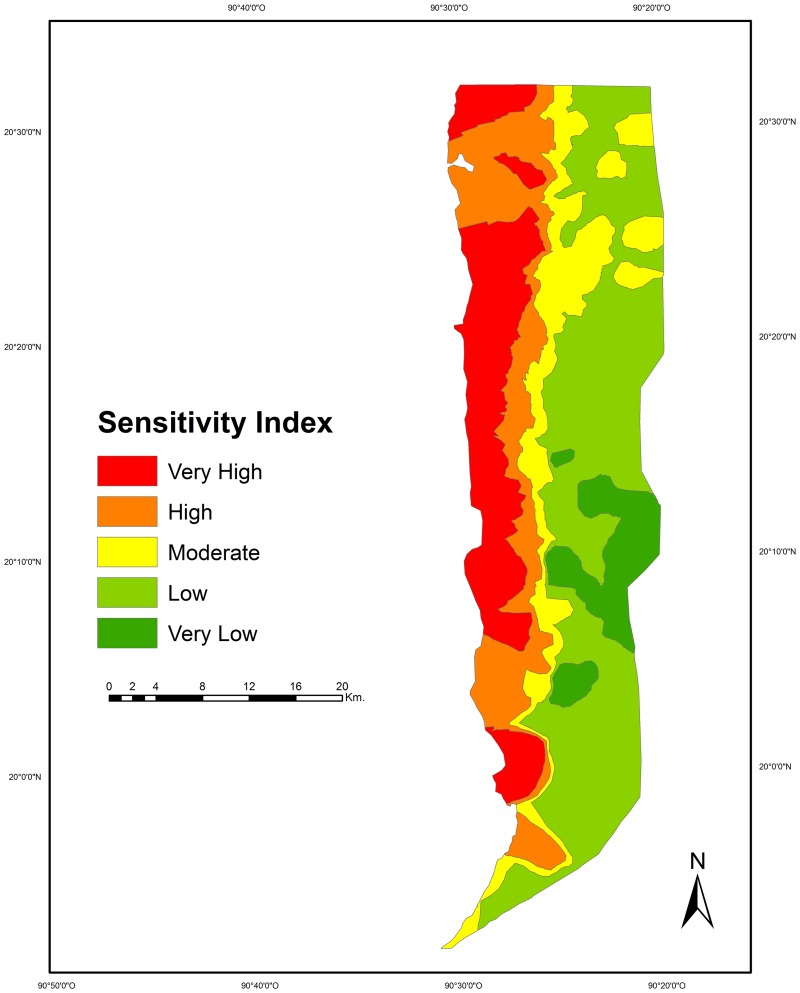
Map of sensitivity to sea level rise due to climate change at Los Petenes Biosphere Reserve.

The assessment of the sensitivity criterion showed that the highest proportion of the LPBR corresponded to the moderate risk category for the indicators “hammock canopy height” (94.6% scored 2, 4.8% scored 1, 0.6% scored 3) and “type of hammock” (46.7% scored 2, 44.1% scored 3, 9.2% scored 1). On the other hand, a higher proportion (49.4%) corresponded to the highest risk category (score 3) of the “patch size” indicator, followed by the low (41.9%, score 1), and moderate (8.7%, score 2) categories. The interpolation of the sensitivity indicators showed that 8.0% of the study area presented very low sensitivity, 39.5% low, 15.5% moderate, 17.2% high, and 19.8 very high (Figs 3 and 5).

**Fig 6:**
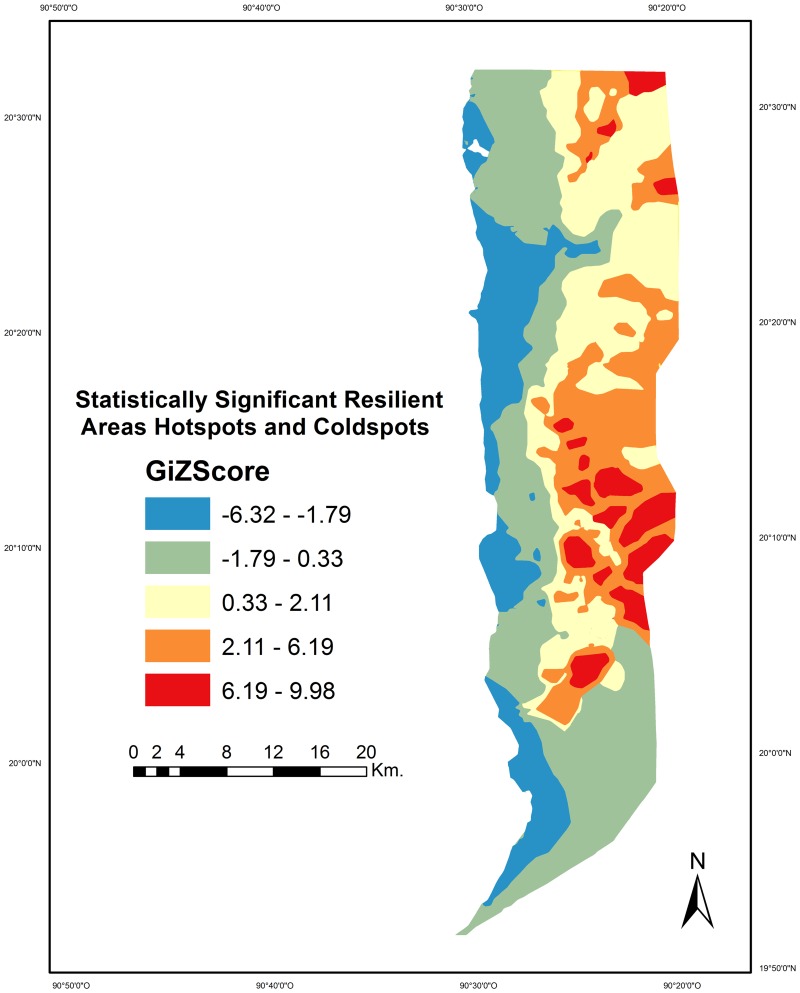
Map of resilient areas at Los Petenes Biosphere Reserve. Statistically significant high values (2.11 to 9.98) represent hot spot areas of resilience and the statistically significant low values (0.33 to -6.32) represent cold spot areas of resilience.

Resilient areas at the LPBR were defined from the interpolation of the estimated persistence value of each hammock polygon. About 65 km^2^ (6.4%) of the LPBR was very highly resilient to SLR due to climate change, and 194 km^2^ (19.1%) was highly resilient (Fig 6). These areas were characterized by being distant from the coastal zone, little direct tidal influence, and consisted of hammocks surrounded by basin mangrove and floodplain forest. These areas also had a low influence of streams and channels, and a low probability of hurricane occurrence. These areas were located in the north-eastern and eastern portions of the LPBR. On the other hand, hammocks with very low resilience covered an area of 172 km^2^ (16.9%) and hammocks with low resilience an area of 346 km^2^ (34.0%). These hammocks were located in coastal areas without vegetation or with scattered mangroves, and with a direct influence of tides and winds. These areas were located in the north-western and southern portions of the LPBR. Areas with medium resilience had dense and high vegetation, they had a very low sensitivity, and high levels of exposure mainly because of a high rate of LCC.

The assessment of the three other weighting schemes (Table 7) showed that high resilient areas had a coincidence of 73.9% with scheme 1, 64.3% with scheme 2, and 72.4% with scheme 3. Since indicators to assess exposure and sensitivity can be quite diverse and that the weighting values assessed gave consistent resilience estimates, these analyses were considered appropriate in accordance with the opinions of experts, published research, and data availability.

## Discussion and Conclusion

A resilience index was developed based on qualitative and quantitative data to assess the magnitude of estimated future SLR disturbances on a coastal ecosystem due to climate change. This type of studies allows a better design of response strategies to face SLR disturbances on sensitive ecosystems, such as hammocks. While a comprehensive resilience analysis would ideally consider the entire ecosystem, this is unrealistic. Nevertheless, every pattern observed in an ecosystem is the result of several functional and evolutionary processes that are simplified or idealized with models [49]. Although models cannot simulate all the intricacies of real ecosystems, these can give a better understanding of the modelled phenomenon, even when several theories might be necessary to explain certain processes [50,51].

Criteria and indicators used in this research were assumed to be appropriate, even though coastal ecosystems may also be sensitive to other variables, such as the increase in sea surface temperature, ocean acidification, salt water intrusion, rising ground water tables, and changing runoff patterns [5], among others. Hammocks persistence could also be affected by rainfall patterns, because precipitation and water seeping into the ground water table tend to dissolve the limestone surface (karst) causing the formation of sinkholes and thus, hammocks. Although it was not possible to assess the rate (not to mention the change in rate) of hammock formation because of changes in precipitation patterns due to climate change, this is a variable that remains to be analysed. The Mexican state of Campeche is located in one of the most vulnerable regions subjected to drought, and this tends to worsen [52,53]. The assessment of precipitation averages projected to 100 years (1980–1999 to 2080–2099) in the Fourth Report on Climate Change of the IPCC [33] estimates a decrease in annual precipitation for the entire Central American region. Some studies have also estimated a decrease in annual precipitation in the Yucatán Peninsula from 10 to 15% and over 30% in the dry and rainy seasons respectively [52,53]. Similarly, Magrin et al. [54] also estimated a decrease between 10 to 22% in annual precipitation by 2090, with periods of drought that could even reach a reduction of 48%. This decrease in rainfall could cause more intense droughts and more frequent forest fires [55], which would further threaten hammocks resilience.

Proximity to coastline is just one of the several factors that affects hammocks resilience. For example, coastal areas in the LPBR are structured by an array of parallel stripes to the coastline due to the presence of fringe mangrove that grows in salt water and where beaches are not present. This suggests a low physical energy from the environment, allowing mangroves to trap sediments and litter [32]. This process creates banks along the coast and estuaries, reducing hammocks exposure in the LPBR and adjacent areas (e. g., Celestún and Isla Arena).

Tidal flooding and large storm surges may play an important role in the vulnerability of hammocks ecosystem; however, the effect of tides was not assessed as an independent indicator. The effect of tides on hammocks was considered as an intrinsic part of other indicators, such as hurricanes impact and type of hammock. Regional studies show that the LPBR area is characterized by its low wave energy and small tides (<50 cm).

This research shows that approximately 25% of the LPBR is highly or very highly resilient to SLR, particularly in areas that are distant from the coast and are only indirectly influenced by tides, have a dense forest cover, and a low probability of hurricane incidence. On the other hand, hammocks with low or very low resilience cover 51% of the LPBR and are characterized by its location near the coastline, are covered with sparse vegetation, and are directly exposed to winds and tides. These results suggest that half of the LPBR is vulnerable to SLR due to climate change.

Although the LPBR is a natural protected area, it does not necessarily grant its conservation. The rate of LCC in some areas is high, particularly in those areas with dense and high vegetation outside the core areas of the LPBR. LCC due to human activities could significantly hamper the potential of inland hammock migration due to SLR as salt water intrudes the coastline.

Finally, the reason for using geostatistical techniques was to add significance to the estimated resilient areas, based on the spatial autocorrelation between adjacent areas. Ordinary Kriging and Getis–Ord G_i_* statistic were considered appropriate to analyse hammocks resilience in the LPBR, to identify the areas that are potentially resilient to flooding by SLR, and also to identify priority conservation areas, as well as areas where mitigation plans should be implemented [46].

This proposed methodological framework to assess resilience against climate change has proven to be efficient even when high-precision data are missing. Lack of data is a fairly common situation in many countries, such as Mexico, where high-precision eco-geographical data are still missing. Resilience analyses in highly vulnerable ecosystems, such as hammocks, are urgently needed because decisions have to be taken to implement the most appropriate adaptation or mitigation strategies, based on the best available information.

## References

[1] March IJ, Cabral H, Echeverria Y, Bellot M, Frausto JM. Adaptación al Cambio Climático en Áreas Protegidas del Caribe de México. Serie Estrategias de Adaptación al Cambio Climático en Áreas Protegidas de México. No. México.; 2011. Report No.: 1.

[2] SEMARNAT-INECC. Quinta Comunicación Nacional ante la Convención Marco de las Naciones Unidas sobre el Cambio Climático México. [Internet]. México, D. F; 2012 Available: http://unfccc.int/resource/docs/natc/mexnc5s.pdf

[3] Espadas C, Conde C, Gay C, Cuanalo C, Larqué A. Atlas escenarios de cambio climático en la Península de Yucatán. 1st ed Unidad de Recursos Naturales C de IC de Y y C de C de la A de la UNA de M, editor. Mérida, Yucatán: Centro de Investigación Científica de Yucatán, A.C; 2009.

[4] Seneviratne SI, Nicholls D, Easterling CM, Goodess S, Kanae J, Kossin Y, et al Changes in climate extremes and their impacts on the natural physical environment. A Special Report of Working Groups I and II of the Intergovernmental Panel on Climate Change (IPCC) In: Field CB, Barros V, Stocker TF, Dahe Q, editors. Managing the Risks of Extreme Events and Disasters to Advance Climate Change Adaptation. Cambridge, UK, and New York, NY, USA: Cambridge University Press; 2012 pp. 109–230.

[5] IPCC. Summary for Policymakers In: Stocker TF, Qin D, Plattner GK, Tignor M, Allen SK, Boschung J, et al, editors. Climate Change 2013: The Physical Science Basis Contribution of Working Group I to the Fifth Assessment Report of the Intergovernmental Panel on Climate Change. 1st ed Cambridge, United Kingdom and New York, NY, USA: Cambridge University Press; 2013 p. 28.

[6] Ortiz M, Méndez A. Escenarios de vulnerabilidad por ascenso del nivel del mar en la costa mexicana del Golfo de México y el Mar Caribe. Investig Geográficas. 1999;39: 68–81. Available: http://www.redalyc.org/articulo.oa?id=56903905

[7] CONANP-SEMARNAT. Programa de Conservación y Manejo Reserva de la Biosfera Los Petenes. 1st ed CONANP, editor. México, D. F: Comisión Nacional de Áreas Protegidas; 2006.

[8] Acosta Lugo E. D, Alonzo Parra M, Andrade Hernández D Castillo Tzab J, Chablé Santo R, Durán C, Espadas Manrique I. Fernández Stohanzlova J. Fraga E, et al Plan de Conservación de la eco-región Petenes—Celestún—Palmar. 1st ed Mérida, Yucatán Pronatura Península de Yucatán, Universidad Autónoma de Campeche; 2010.

[9] Yánez-Arancibia A, Lara-Domínguez A. L, Rojas Galaviz JL, Villalobos Zapata GJ, Rivera Arriaga D, Zárate Lomelí G, et al. Caracterización Ecológica Ambiental y de los Recursos Naturales de la Región de Los Petenes. Campeche; 1996. Report No.: 1198.

[10] Torres Castro IL, Vega Cendejas ME, Schmitter Soto JJ, Palacio Aponte G, Rodiles Hernández R. Ictiofauna de sistemas cárstico-palustres con impacto antrópico : los petenes de Campeche, México. Rev Biol Trop. 2009;57: 141–157. 0034–7744 19637696

[11] The Nature Conservancy (TNC), editor. Guía para la elaboración de programas de adaptación al cambio climático en áreas naturales protegidas. 1st ed México: Comisión Nacional de Áreas Naturales Protegidas-Fondo Mexicano para la Conservación de la Naturaleza A.C; 2011.

[12] Borrini-Feyerabend G. Dudley T, Jaeger B, Lassen N, Pathak Broome A, Sandwith PT. Governance of Protected Areas: From understanding to action. 20th ed Gland, Switzerland: IUCN: International Union for Conservation of Nature and Natural Resources; 2013.

[13] Brown K. Policy discourses of resilience In: Pelling M et al, editor. In Climate Change and the Crisis of Capitalism: A Chance to Reclaim, Self, Society and Nature. Routledge; 2012 pp. 37–50. Available: http://www.scopus.com/record/display.url?eid=2-s2.0-84891838514&origin=inward&txGid=3C7A907E9628FA002A538578D8D5B1C0.iqs8TDG0Wy6BURhzD3nFA%3a2#

[14] (World Bank). Pilot Program for Climate Resilience under the Strategic Climate Fund, World Bank [Internet]. 2008. Available: http://www.climateinvestmentfunds.org/cif/home

[15] Nimmo DG, Mac Nally R, Cunningham SC, Haslem a., Bennett a. F. Vive la résistance: reviving resistance for 21st century conservation. Trends Ecol Evol. Elsevier Ltd; 2015;30: 516–523. 10.1016/j.tree.2015.07.008 26293697

[16] Brand FS, Jax K. Focusing the Meaning(s) of Resilience: Resilience as a Descriptive Concept and a Boundary Object. Ecol Soc. 2007;12: 16 Available: http://www.ecologyandsociety.org/vol12/iss1/art23/

[17] Holling CS. Resilience and stability of ecological systems. Annu Rev Ecol Syst. 1973;4: 23.

[18] Gallopín GC. Linkages between vulnerability, resilience, and adaptive capacity. Glob Environ Chang. 2006;16: 293–303. 10.1016/j.gloenvcha.2006.02.004

[19] Folke C. Resilience: The emergence of a perspective for social–ecological systems analyses. Glob Environ Chang. 2006;16: 253–267. 10.1016/j.gloenvcha.2006.04.002

[20] Pimm SL. The complexity and stability of ecosystems. Nature. 1984;307: 321–326.

[21] Connell JH, Sousa W. On the evidence needed to judge ecological stability or persistence. Am Nat. 1983;121: 789–824.

[22] Gunderson LH. Ecological Resilience. In Theory and Application. Annu Rev Ecol Syst. 2000;31: 425–439. Available: http://www.jstor.org/stable/221739.

[23] Turner BL, Kasperson RE, Matson P a, McCarthy JJ, Corell RW, Christensen L, et al A framework for vulnerability analysis in sustainability science. Proc Natl Acad Sci U S A. 2003;100: 8074–9. 10.1073/pnas.1231335100 12792023 PMC166184

[24] Ludwig D, Walker B, Holling CS. Sustainability, Stability, and Resilience. Ecol Soc. 1997;1: 1–24. Available: http://www.consecol.org/vol1/iss1/art7/

[25] Gunderson L, Holling CS, editors. Panarchy: Understanding Transformations in Human and Natural Systems. 2nd ed Washington D.C: Island Press; 2002.

[26] Carpenter S, Walker B, Anderies JM, Abel N. From Metaphor to Measurement: Resilience of What to What? Ecosystems. 2001;4: 765–781. 10.1007/s10021-001-0045-9

[27] Hodgson D, McDonald JL, Hosken DJ. What do you mean, “resilient”? Trends Ecol Evol. Elsevier Ltd; 2015;30: 503–506. 10.1016/j.tree.2015.06.010 26159084

[28] Kates RW. Climate Impact Assessment: Studies of the Interaction of Climate and Society. 1st ed Kates RW, Ausubel J, Berberian M, editors. Worcester, Massachusetts: Wiley, New York; 1985.

[29] Eakin H, Luers AL. Assessing the Vulnerability of Social-Environmental Systems. Annu Rev Environ Resour. 2006;31: 365–394. 10.1146/annurev.energy.30.050504.144352

[30] Brand F. Ecological Resilience and its Relevance within a Theory of Sustainable Development. Leipzig-HalleLeipzig-Halle; 2005. Report No.: 0948–9452.

[31] Anderson KE, Cahoon D, Gesch D, Gill S, Gutierrez B, Thieler R, et al Coastal Sensitivity to Sea Level Rise: A Focus on the Mid-Atlantic Region. Washington DC; 2009.

[32] Ramieri E, Hartley A, Barbanti A, Santos FD, Gomes A, Laihonen P, et al. Methods for assessing coastal vulnerability to climate change [Internet]. Bologna, Italy; 2011. Report No.: 1. Available: http://cca.eionet.europa.eu/

[33] Lehner B, Verdin K, Jarvis A. HydroSHEDS. Technical Documentation. In: World Wildlife Fund US, Washington, DC. 2006.

[34] Homogeneous A. NEXTMap ^®^ World 30 ^™^ Meter digital surface model. 2012.

[35] Tighe ML. Validation of a new 30 meter ground sampled global DEM using ICESat Lidar elevation reference data [Internet]. 2012 [cited 10 Sep 2014]. Available: http://blackbridge.com/rapideye/upload/INTERMAP_NEXTMap_World_30_LTR_English.pdf

[36] INEGI. Carta de uso del suelo y vegetación Serie V. In: Instituto Nacional de Estadística y Geografia [Internet]. Mexico: Instituto Nacional de Estadística y Geografía (INEGI); 2013 [cited 17 Jun 2014]. Available: http://www3.inegi.org.mx/sistemas/productos/

[37] NOAA. Historical Hurricanes Tracks v4.0. In: National Oceanic and Atmospheric Administration. [Internet]. 2013 [cited 20 Jun 2014]. Available: http://coast.noaa.gov/hurricanes/?redirect=301ocm 23888591

[38] IPCC. Impacts, Adaptation and Vulnerability: Working Group II contribution to the Fourth Assessment Report of the IPCC In: Parry ML, Canziani OF, Palutikof JP, Van Der Linden PJ, Hanson CE, editors. Climate Change 2007. 1st ed New York: Cambridge University Press; 2007.

[39] Nerheim S. Growing demands for downscaling of climate information; examples from predictions of future sea levels. 6th US/EU-Baltic International Symposium, Ocean Observations, Ecosystem-Based Management and Forecasting. Tallin, Estonia: Marine Systems Institute at Tallinn University of Technology (MSI) (EST), Institute of Electrical and Electronics Engineers (IEEE), Oceanic Engineering Society (IEEE/OES); 2008. 10.1109/BALTIC.2008.4625543

[40] Church JA, Clark A, Cazenave JM, Gregory S, Jevrejeva A, Levermann MA, et al Sea Level Change In: Stocker T.F., Qin D., Plattner G.-K., Tignor M., Allen S.K., Boschung J., Nauels A., Xia VB Y. and PMM, editor. Climate Change 2013: The Physical Science Basis Contribution of Working Group I to the Fifth Assessment Report of the Intergovernmental Panel on Climate Change. 1st ed Cambridge, United Kingdom and New York, NY, USA: Cambridge University Press; 2013 p. 1137.

[41] Herrera-Silveira J, Ciau-Cardozo E, Toyohiko M, Tsuruda K. Manual Práctico para la rehabilitación del ecosistema de manglares en Yucatán, México. 3rd ed JICA, CONANP Cinvestav, Reserva de la Biosfera Ría Celestún, editors. Yucatán; 2010.

[42] Karl TR, Meehl G a, Miller CD, Hassol SJ, Waple A, Murray W. Weather and Climate Extremes in a Changing Climate Regions of Focus: North America, Hawaii, Caribbean, and U.S. Pacific Islands. Synth Assess Prod 33. 2008;3.3: 164 Available: http://www.climatescience.gov/Library/sap/sap3-3/final-report/default.htm

[43] Schott T, Landsea C, Hafele G, Lorens J, Thurm H, Ward B, et al. The Saffir-Simpson Hurricane Wind Scale. In: NOAA/ National Weather Service [Internet]. 2012 pp. 1–4. Available: http://www.nhc.noaa.gov/pdf/sshws.pdf

[44] (AOML/NOAA). “La Escala Saffir/Simpson para Huracanes.” In: Atlantic Oceanographic & Metereological Laboratory [Internet]. 2006. Available: http://www.aoml.noaa.gov/general/lib/laesca.html

[45] Eastman JR. IDRISI Kilimanjaro Guide to GIS and Image Processing. Worcester, Massachusetts: Clark University; 2009.

[46] Cintron G, Schaeffer-Novell Y. The Mangrove Ecosystem: Research Methods Sneadaker SC, Sneadaker JG, editors. Methods for studying mangrove structure. Paris: UNESCO; 1984.

[47] Posada G, Ruiz G, Vega B, Silva R, G. V, Pulido A. Determinación de Marea Astronómica y Meteorológica mediante una Red de Sensores Mareográficos para el Estado de Campeche XVI Congreso Nacional de Oceanografía. Ensenada, Baja California, México; 2010.

[48] Posada G, Durán G, Silva R, Maya ME, Salinas J. Vulnerability to Coastal Flooding Induced by Tropical Cyclones International Conference on Coastal Engineering. Shangai, China; 2010 2156–1028

[49] McGarigal K, Marks BJ. FRAGSTATS Spatial pattern analysis program for quantifying landscape structure. Oregon State University; 1994.

[50] Lopez R, Heggem D, Edmonds C, Jones B, Bice L, Hamilton M, et al A Landscape atlas of ecological vulnerability: Arkansas’ white river watershed and the Mississippi alluvial valley Ecoregion. Arkansas; 2003.

[51] Michener W, Blood E, Bildstein K, Brinson M, Gardner L. Climate change, hurricanes, tropical storms and rising sea level in coastal wetlands. Ecol Appl. 1997;7: 770–801.

[52] Saaty TL. A scaling method for priorities in hierarchical structures. J Mathematical Psychology. 1977 pp. 234–281.

[53] Sánchez O, Islebe G. Hurricane Gilbert and structural changes in a tropical forest in South-Eastern Mexico. Glob Ecol Biog. 1999;8: 29–38.

[54] Koyoc-Ramirez LG, Mendoza-Vega J, Pérez Jimenez JC, Torrescano-Valle N. Efectos de la perturbación antrópica en petenes de selva en Campeche, México. Acta Bot Mex. 2015;110: 89–103.

[55] Diggle P, Ribeiro P. Model-Based Geostatistics. Springer New York; 2007.

[56] Order JK, Getis A. Local spatial autocorrelation statistics: Distributional issues and an application. Geogr Anaysis. 1995;27: 286–306.

